# Efficacy of interventional radiology in the management of portal hypertension: A narrative review

**DOI:** 10.1097/MD.0000000000030018

**Published:** 2022-08-19

**Authors:** Toru Ishikawa

**Affiliations:** Department of Gastroenterology, Saiseikai Niigata Hospital, Niigata, Japan.

**Keywords:** balloon-occluded retrograde transvenous obliteration, interventional radiology, partial splenic embolization, percutaneous transhepatic obliteration, portal hypertension, transjugular intrahepatic portosystemic shunting, transjugular retrograde obliteration of gastric varices

## Abstract

Portal hypertension is associated with numerous adverse effects, including the formation of gastroesophageal varices and a portal vein general circulation shunt. Portal hypertension can lead to portal blood flow into the liver and a subsequent reduction in liver function. Clinical interventions can be hampered by a concurrent reduction in circulating platelets associated with increased splenic activity. Pharmaceutical interventions for the treatment of complications associated with portal hypertension have achieved various degrees of success. However, an effective therapeutic strategy for portal hypertension has not yet been established. A literature search was performed using “PubMed.” Database between 1966 and January 2021 using the following keywords: portal hypertension, interventional radiology, balloon-occluded retrograde transvenous obliteration, transjugular retrograde obliteration of gastric varices, percutaneous transhepatic obliteration, partial splenic embolization, and transjugular intrahepatic portosystemic shunting. In this narrative review, we summarize the application of interventional radiology in patients with portal hypertension, including techniques for embolization of collateral veins and portal pressure reduction. These up-to-date interventional radiology techniques can be used to treat portal hypertension. The data that support the findings of this study are available from the corresponding author, upon reasonable request.

**Lay summary**

Portal hypertension is the most common cause of chronic liver disease. Pharmacotherapy for portal hypertension has recently progressed. However, some patients continue to have uncontrolled portal hypertension despite pharmacological therapy. Herein, we describe the techniques of interventional radiology for the treatment of portal hypertension.

**Key points:**

• Portal hypertension is most commonly caused by chronic liver disease.
• Effective therapies to treat portal hypertension are lacking.
• Interventional radiology (IVR) techniques can be used for portal hypertension.
• IVR can achieve embolization of collateral veins and portal pressure reduction.
• It is a minimally invasive therapy for portal hypertension.

## 1. Introduction

The changes in hemodynamics due to cirrhosis lead to adverse effects, including complications of portal hypertension, such as the formation of gastroesophageal varices and portosystemic shunt. The prognosis of patients with cirrhosis progressively deteriorates with the cumulative occurrence of ascites, variceal hemorrhage, hepatic encephalopathy (HE), and spontaneous bacterial peritonitis.^[1]^ Portal hypertension can lead to a reduction in portal blood flow into the liver and subsequent reduction in liver function. Portal blood flow into the liver due to portal hypertension impairs hepatic functional reserve. Pharmacological treatments for complications of portal hypertension have recently been developed.^[2]^ Although these advanced pharmacological therapies have been shown to be useful for complications associated with portal hypertension, therapeutic strategies for portal hypertension are also important.^[3]^ Interventional radiology (IVR) is an image-guided treatment method with minimal invasiveness that is performed using a catheter or needle under the guidance of X-ray fluoroscopy or computed tomography (CT), ultrasound (US), and magnetic resonance imaging can be used as guide for IVR procedures as well. As IVR is minimally invasive, the burden on the patient’s overall health is small, and its therapeutic effect is comparable to that of surgery.

Therapeutic strategies for portal hypertension should improve overall survival.^[4–7]^ The modification of blood flow achieved via IVR in patients with portal hypertension improves the pathological alterations associated with portal hypertension. This review aims to provide an overview of the various IVR treatment methods for portal hypertension and provide insight into these emerging therapies and future therapeutic options.

## 2. Methods

We searched the PubMed database between 1966 and January 2021 using the following keywords: portal hypertension, IVR, balloon-occluded retrograde transvenous obliteration (B-RTO), transjugular retrograde obliteration of gastric varices (TJO), percutaneous transhepatic obliteration (PTO), partial splenic embolization (PSE), and transjugular intrahepatic portosystemic shunting.

Concerning about the ethical issues, we declared that this work does not require ethical approval because it is a literature review of human and animal experimental and clinical ethics.

### 2.1. IVR for portal hypertension

Various IVR techniques are available for the treatment of portal hypertension complications. In a normal liver, venous return from visceral organs flows into the liver via the portal trunk (hepatopetal blood flow). As liver damage causes increased portal pressure, some veins become congested and the direction of blood flow within the veins changes (hepatofugal blood flow), and collateral blood flow (portosystemic shunts) occurs to reduce portal pressure.

In chronic liver disease, as intrahepatic vascular resistance increases (backward flow theory) and collateral veins develop, increased blood flow into the portal venous system is required to maintain portal hypertension (forward flow theory). Therefore, embolization of the collateral veins and/or reduction in portal pressure is required.

IVR is used to treat portal hypertension via embolization of the collateral veins and reduction of the portal pressure. The techniques used to embolize collateral veins include B-RTO, TJO, and PTO. The techniques used to reduce portal pressure include PSE, transjugular intrahepatic portosystemic shunt (TIPS), and portal vein stents for secondary portal hypertension secondary to portal vein tumor embolization.

#### 2.1.1. Embolization of the collateral veins.

IVR embolization can be categorized based on the direction of blood flow as retrograde (B-RTO and TJO) or anterograde (PTO).

##### 2.1.1.1. B-RTO and TJO.

Surgical treatment, such as Hassab surgery, is the standard treatment for solitary gastric varices, as endoscopic management is less effective for the treatment of gastric varices, which have a high blood flow volume, than the treatment of esophageal cancer.^[8]^

Furthermore, endoscopic sclerotherapy may lead to several complications, including venous and systemic thromboembolism (such as pulmonary embolism or stroke), ulcers, protracted bleeding, or splenic and portal vein thrombosis.^[9]^ However, since B-RTO was first reported by Kanagawa et al^[10]^ in 1991, it has become the standard treatment for isolated gastric varices as it eliminates the varices and results in favorable therapeutic outcomes. This procedure involves retrograde cannulation of the outflow channels, draining the gastric varices through the femoral or jugular vein, and the obliteration of the varices and collateral veins via balloon occlusion, followed by coils and sclerosant.^[10]^

B-RTO is indicated in patients with gastric varices with a history or risk of rupture with a gastrorenal shunt or an inferior phrenic vein that drains directly into the inferior vena cava and in patients with HE due to these shunts. Hirota et al^[11]^ created a classification system for hemodynamics in these patients; however, shunt vessels have various subtypes of hemodynamics and are often difficult to treat owing to their anatomical diversity.^[12]^ In addition to the wide variety of inflow channels, accessory outflow channels and main outflow channels are also present. When catheterization is not possible in the main drainage vein of gastric varices or gastric varices with a direct connection to the coronary vein or esophageal varices, treatment with the B-RTO procedure is difficult.^[13]^ To overcome these challenges, maximum intensity projection imaging of contrast-enhanced CT that matches the portal vein phase prior to B-RTO surgery can be used.^[14]^ The catheter-placement time of the B-RTO approach via the transfemoral approach has been reported to be 30 minutes.^[10]^

In 1996, Chikamori et al^[15]^ reported TJO, which is a 24-hour catheter placement technique using a transjugular vein approach. TJO is one type of B-RTO. Preoperative diagnostic imaging is important for selecting an appropriate approach.

**Figure 1.**
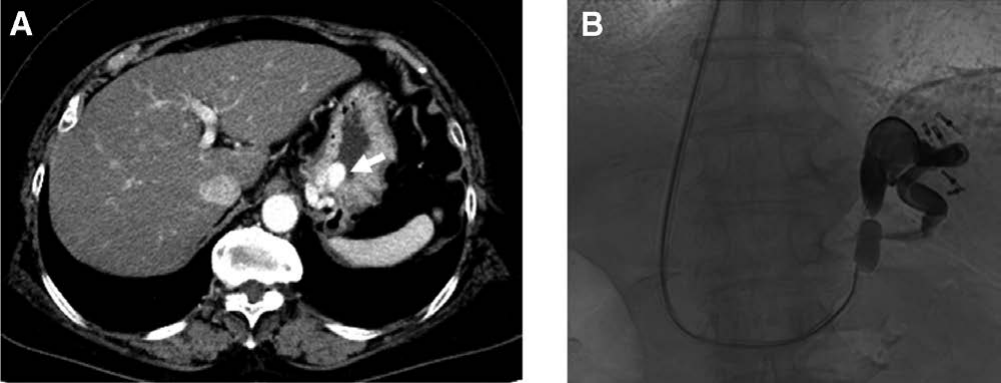
(A) Axial CT images acquired at portal venous phase demonstrating large fundal GV (arrow). (B) Fluoroscopy, showing partial thrombosis of the gastric varices. The gastric varices and gastrorenal shunt are fully opacified by the sclerosant with contrast medium during TJO. CT = computed tomography, GV = gastric varices, TJO = transjugular retrograde obliteration of gastric varices.

A representative case is shown in Figure 1. The technically, successful B-RTO rate was 91% (79%–100%).^[16]^ Adverse events associated with B-RTO include fever, chest pain, gastrointestinal symptoms, hemoglobinuria, ascites, and pleural effusion. It was shown that occlusion of a large gastrorenal shunt could increase the hepatic venous pressure gradient by up to 44% from the baseline. B-RTO was found to aggravate preexisting esophageal varices (ranging from 30% to 68%), leading to variceal bleeding even though associated death has never been reported.^[17]^

##### 2.1.1.2. PTO.

When the deterioration of liver function is severe, the increase in portal hypertension shunts blood flow, increasing the volume of blood that does not pass through the liver and, resulting in HE (also termed shunt encephalopathy).^[18,19]^ Conservative treatment, such as the administration of branched-chain amino acids and lactulose, stabilizes the symptoms in most patients.^[20,21]^ However, if the volume of shunted blood is significantly increased, conservative treatment only temporarily improves the state of consciousness, and persistent hyperammonemia may lead to altered consciousness.

The presence of a shunt increases the bioavailability of intestinal ammonia and the risk of HE. Several clinical and pathophysiological studies have suggested the importance of portal-systemic shunts in the development of HE. Spontaneous portosystemic shunts were identified in 71% of the patients with cirrhosis and chronic HE refractory to standard medical treatments.^[22]^

However, although shunt obstruction may improve HE, it leads to an increase in portal pressure, which may result in complications such as exacerbation of gastrointestinal varicose veins, ascites, and portal vein thrombosis.^[23]^ These must be considered when performing PTO.

PTO was first reported by Lunderquist and Vang^[24]^ in 1974 and has been widely used as emergency treatment for patients with bleeding varicose veins. Before PTO, US was performed in all patients to determine the best access route to the portal venous system. US is an inpatient procedure that requires conscious sedation. Percutaneous transhepatic puncture of the intrahepatic branch of the portal vein was achieved using an 18-gauge needle under sonographic guidance. A 5-French gauge sheath catheter was introduced into the portal vein. Direct portography was performed to identify the feeding and draining veins of the gastric varices or the shunt veins. Gastric varices or shunt veins often have multiple feeding veins, and a coaxial catheter is inserted into these accessory feeding veins. Feeding veins were embolized using microcoils or sclerosing agents.^[25]^

The development of a new blood supply path after embolization and recurrent or rebleeding varicose veins is a challenge to the PTO procedure.^[24]^ PTO is typically performed using an anterograde approach and is slightly invasive, as it involves percutaneous and transhepatic approaches. However, as it is an anterograde, it is relatively easy to understand hemodynamics via contrast examination.

**Figure 2.**
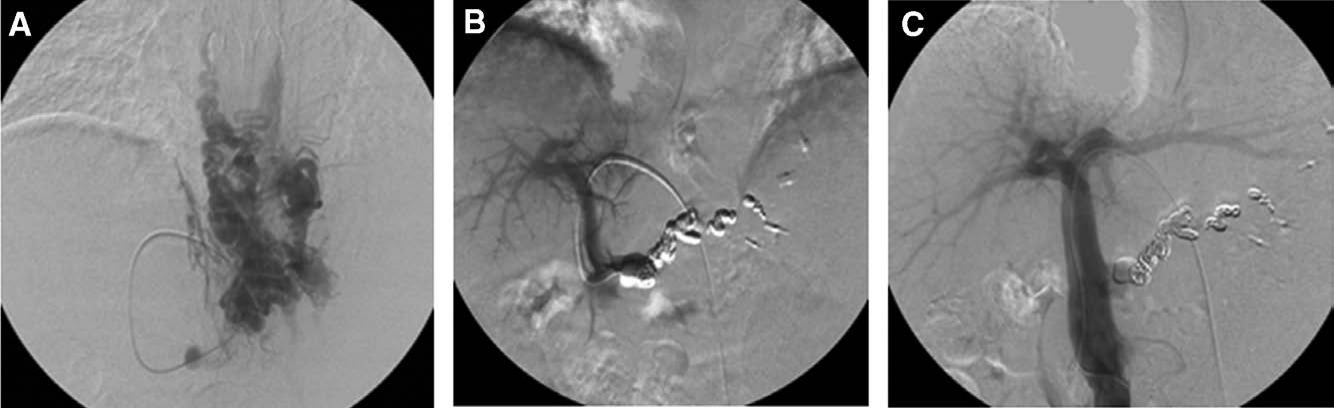
(A) Percutaneus transhepatic portography shows from the left gastric vein to the collateral veins including paraesophageal vein, which caused esophageal varices hemorrhage. (B) The feeding veins were embolized with microcoils and a sclerosing agent. (C) Percutaneous transhepatic portography after treatment showing that the varix and its feeder were embolized.

Patients with HE due to portal hypertension associated with refractory esophagogastric varices had improved Child-Pugh scores 3 months after PTO compared to baseline.^[25]^ PTO maintain the functional hepatic reserve in patients with gastroesophageal shunts, B-RTO-refractory HE without gastrorenal shunts, and gastric varices.^[25]^ A representative case is shown in Figure 2.

#### 2.1.2. Portal pressure reduction.

##### 2.1.2.1. Partial splenic embolization.

Maddison^[26]^ first reported splenic artery embolization in 1973; however, the indications were initially limited because of serious complications, including splenic abscess and pneumonia sepsis. However, in 1979, Spigos et al^[27]^ reported PSE with a limited infarct area, which improved safety and has been widely applied in the clinic.

In the PSE procedure, a percutaneous catheter was inserted into the right femoral artery under local anesthesia with 1% lidocaine. The tip of the catheter was then advanced into the hilum of the splenic artery. Branches of splenic arteries were embolized using microcoils and gelatin sponges. The upper branch of the splenic artery was left untreated, and the final embolization rate was approximately 70%.^[28]^

PSE is indicated in patients with splenic hypertension and aims to improve thrombocytopenia, esophageal/gastric varices due to portal hypertension, and portal hypertensive gastritis.^[29]^ The improvement of hepatic function is thought to be due to the improvement of hepatic blood flow, PSE has been reported as an alternative treatment to splenectomy as it is relatively minimally invasive and effective^[30,31]^; however, skill is required to ensure the intended infarct rate. Infarct rates less than 50% have been associated with recurrent splenic hypertension, whereas infarct rates greater than 50% have been associated with increased complications.^[32]^ Determining the appropriate infarct rate is extremely important, and several studies have reported that it is typically 60%–80%.^[33,34]^ However, digital subtraction angiography imaging and detailed intraoperative CT imaging are required to evaluate the infarct rate. Cone-beam CT should also be used to intraoperatively measure infarction rate.^[35]^

In patients with cirrhosis, hypersplenism and decreased platelet counts occur with the progression of liver fibrosis.^[36]^ Hepatocellular carcinoma is often associated with liver cirrhosis, and its treatment methods range from surgery, local coagulation therapy, transcatheter arterial chemoembolization therapy, systemic chemotherapy, and liver transplantation; however, thrombocytopenia during treatment is a major challenge.^[37]^

It has been reported that simultaneous transcatheter arterial chemoembolization and PSE in patients with hepatocellular carcinoma with thrombocytopenia can maintain the hepatic reserve.^[38]^

Compared with splenectomy, PSE preserves the function of the spleen and is minimally invasive, with a low rate of portal vein thrombosis. However, serious complications including fever, abdominal pain/vomiting, ascites/pleural effusion, and splenic abscess/peritonitis have been reported, and the indications for PSE should be carefully considered.^[39]^

Patients with myelosuppression who have been treated with anticancer or immunosuppressive drugs are at a risk of immunosuppression. Postsplenectomy sepsis and overwhelming postsplenectomy infections have a fatality rate of over 70% and are more likely to occur after splenectomy than after PSE.^[40]^ Splenic dysfunction can occur after PSE. The appearance of Howell-Jolly bodies (HJBs) in peripheral erythrocytes is a marker of hyposplenism.^[41]^ HJBs are inclusion bodies within red blood cells that stain with May-Giemsa stain and do not appear in normal individuals. They are found in patients with blood disorders, after splenectomy, or in a functionally asplenic state. A low residual splenic volume may account for the presence of HJBs after PSE.^[42]^ In such cases, patients are considered immunosuppressed, and measures such as the administration of pneumococcal vaccines are required.

##### 2.1.2.2. Transjugular intrahepatic portosystemic shunt.

Rösch et al^[43]^ first proposed TIPS using animal experiments in 1969. In January 1981, Colapinto et al^[44]^ conducted TIPS using balloon dilatation for the first time in a clinical study. The first TIPS performed in humans using metal stents was performed in 1988.^[45]^

Yamada^[46]^ first performed TIPS in Japan in 1992, treating patients with bleeding esophageal varices using the Rosch-Uchida transjugular liver access set (Cook Medical, Bloomington, IN). TIPS is considered the culmination of IVR procedures, as it involves puncture, balloon dilation, and stent placement.

The TIPS procedure was performed under guidance of digital subtraction angiography. In summary, venous access was attained via the right internal jugular vein, and the right or middle hepatic vein was catheterized. A standard Rösch-Uchida TIPS set (Cook Medical) was used to create a parenchymal tract between the hepatic vein and intrahepatic portions of the portal vein. In some patients in whom access to the portal vein by transhepatic puncture was difficult, a 0.014-inch wire was percutaneously inserted into the portal system to provide access. After measurement of the portal vein and right atrial pressures, the tract was dilated using balloon catheters, and a bare stent (Bard E-Luminexx Vascular Stent, C. R. Bard, Inc, Karlsruhe, Germany) was deployed, followed by a stent graft (Viabahn, W. L. Gore & Associates, Inc, Flagstaff, AZ) to line the tract. The stents were typically 8 mm in diameter. The length of the bare stent was selected according to the length from the entry site in the portal vein to the inferior vena cava, plus 1–2 cm. The added length of the bare metal stent was placed in the portal vein. The covered stents were 5 or 10 cm in long, with the distal portion extending slightly into the portal vein. The portosystemic pressure gradient was measured after the creation of portosystemic shunts. Technical success of TIPS is defined as the successful creation of a shunt between the hepatic and intrahepatic branches of the portal vein.^[47,48]^

Indications for TIPS include refractory hepatic ascites that are unresponsive to diuretics, albumin administration, and salt intake restriction and require fine needle drainage, refractory esophageal varices with repeated bleeding even after endoscopic treatment, and portal hypertension gastroenteropathy. Contraindications of TIPS include severe or refractory HE, severe cardiopulmonary diseases, such as severe pulmonary hypertension and congestive heart failure, presence of tumors and cysts in the TIPS puncture pathway, hepatorenal failure, and sepsis.^[49]^

**Figure 3.**
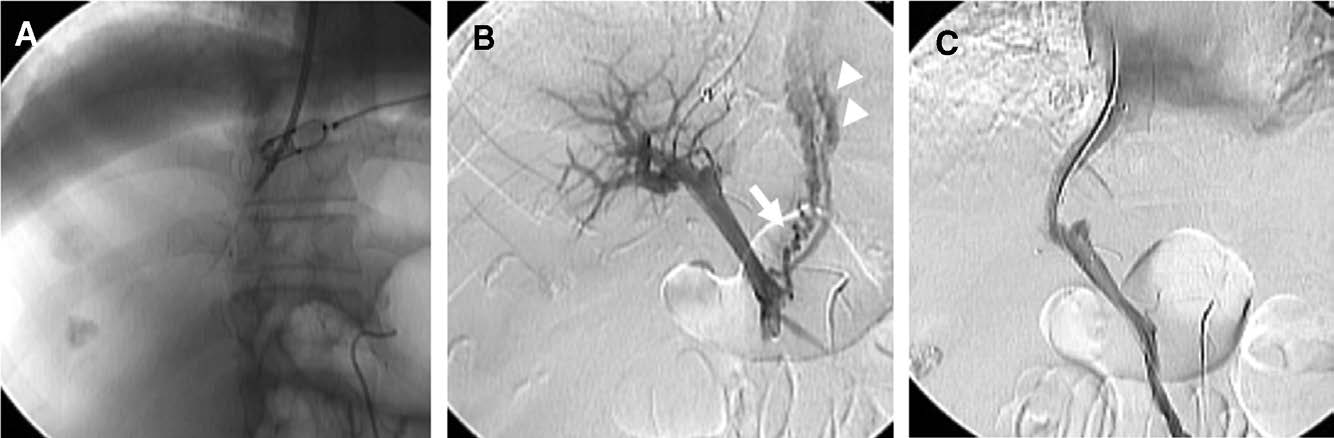
(A) The 3D roadmap is fused with the working fluoroscopy screen. (B) Digital substraction portal venogram prior to TIPS procedure depicts a left gastric vein (arrow) supplying esophageal varices (arrowhead). (C) Abdominal radiograph after the TIPS procedure. Esophageal varices are no longer visualized. TIPS = transjugular intrahepatic portosystemic shunt.

Puncture from the hepatic vein to the portal vein is the most difficult step in the TIPS procedure. Therefore, it is important to obtain preoperative contrast 3D CT images to understand the patient’s vascular anatomy, use an intraoperative intrahepatic artery guide wire to determine the position of the Glisson capsule, and use intraoperative fluoroscopy to view the anterior and lateral aspects of the surgical field. The frequency of puncture is low but may result in intra-abdominal or biliary bleeding during surgery.^[49]^ HE may occur after TIPS, although most cases of HE due to TIPS can be controlled with medical treatment. Stenosis or occlusion of the TIPS shunt can be treated via vasodilation with a percutaneus transluminal angioplasty balloon catheter or the placement of an additional stent.^[50]^ A representative case is shown in Figure 3. The success rate of TIPS ranges from 67% to 100% in 19 case series.^[51]^ Shunt dysfunction following TIPS placement is a major problem.^[52]^

In the era of bare metal stents, TIPS dysfunction was a major problem that led to relatively low primary patency rates, typically less than 50% at 1 year.^[53]^ More recently, covered stent-grafts have been shown to improve TIPS patency of TIPS. Expanded polytetrafluoroethylene-covered TIPS has improved patency rates and clinical outcomes compared with bare metal TIPS. The primary patency rates at 2 years have been shown to range from 62% to 89%.^[54,55]^

Other major complications described in the literature include hemoperitoneum, stent malpositioning, hemobilia, hepatic infarction, and resistant HE. Minor complications included biliary duct puncture, gallbladder puncture, right kidney puncture, transient pulmonary edema, transient HE, and transient renal failure. Such complications may occur in 4%–8% of cases.^[51,56]^

Although there are the above complications, it is unclear when TIPS is performed.

A randomized control trial confirmed that the early use of TIPS with an expanded polytetrafluoroethylene-covered stent was associated with significant reductions in the failure to control bleeding, rebleeding, and mortality, with no increase in the risk of HE.^[57]^

It is also necessary to verify the TIPS timing in larger study.

##### 2.1.2.3. Portal vein stenting.

Esophagogastric varices associated with portal vein tumor thrombosis in hepatocellular carcinoma are refractory to pharmacological treatments.^[58]^ Stents have been reported as effective treatments for portal vein stenosis in patients with portal vein malignancies,^[59]^ and they are believed to be effective for portal vein tumor embolic hepatocellular carcinoma.

The left branch of the portal vein or right branch of the portal vein was selected under ultrasonic guidance and punctured with a puncture needle. Although flexion of the umbilical region is difficult to navigate during sheath insertion, the left branch of the portal vein has a short distance to the portal vein branch. The right branch of the portal vein has a straight route to reach the main trunk of the portal vein after portal vein puncture; however, there is a high risk of bleeding during puncture and sheath removal when ascites is present. The sheath was inserted into the intrahepatic portal vein branch and passed through portal vein stenosis along the guide wire. An angiographic catheter was inserted, and portal vein imaging was performed before stent deployment. The stent placed in the portal vein stenotic region has the same diameter as the normal portal vein or is 1–2 mm larger. The target length was at least 1 cm beyond the obstruction length in both the directions. Vasodilation was performed using a balloon catheter. After the stent was placed, portal vein imaging was performed, and the sheath was removed with the catheter while embolizing the puncture route with embolic material.

Complications associated with puncture during the placement of portal vein stents include subcutaneous bleeding, liver injury, hemobilia, hepatic artery injury, and intraperitoneal bleeding.^[60]^ In addition, abdominal pain, back pain, and fever may also occur.

Approximately 20% of extrahepatic portal vein stenosis or occlusion is due to malignant portal vein stenosis, caused by hepatocellular carcinoma or pancreatic cancer.^[61]^

Yamakado et al^[59]^ reported an average patency of 12.4 months and an average survival of 13.7 months in patients with portal vein-infiltrating hepatocellular carcinoma in whom portal vein stents were placed. Portal vein stent placement may be useful as a prognostic factor in preventing esophagogastric variceal bleeding due to portal vein tumor embolism.

In patients in whom portal blood flow is reduced due to portal vein infiltration, it is often impossible to continue treatment for hepatocellular carcinoma due to prolonged jaundice or the progression of liver failure, resulting in no life-prolonging effect of portal vein stents. If hepatic blood flow is improved by the portal vein stent, the hepatic reserve is also improved, liver failure is avoided, and the treatment for hepatocellular carcinoma can continue.^[62]^ The appropriate timing of portal vein stent placement in patients with cancer requires further verification in studies with large patient populations.

## 3. Conclusion

Pharmacological therapies for patients with portal hypertension have evolved, and the number of treatment options has increased in recent years. However, the addition of IVR treatment is expected to improve the prognosis of some patients with portal hypertension.^[63]^ Surgical intervention should also be considered as one of the safest techniques currently available for portal hypertension. Laparoscopic surgery is a less invasive method than open surgery; however, laparoscopic surgery for portal hypertension is still considered a high-risk operation, with collateral venous change and severe splenomegaly and a bleeding tendency.^[64]^

Liver transplantation is the only curative treatment for portal hypertension in patients with end-stage liver disease. Patients with good liver function despite portal hypertension may be managed satisfactorily without undergoing liver transplantation. However, liver transplantation should be considered in patients with end-stage liver disease. Patients on the waiting list need symptomatic “bridging therapy,” such as IVR, until liver transplant is available.

It is important to determine the treatment options that are indicated for each pathological condition. Further research regarding the use of IVR in patients with portal hypertension is required.

## Acknowledgments

We would like to thank Editage (www.editage.com) for the English language editing.

## Author contributions

Conceptualization: Toru Ishikawa.

Data curation: Toru Ishikawa.

Formal analysis: Toru Ishikawa.

Investigation: Toru Ishikawa.

Methodology: Toru Ishikawa.

Writing – original draft: Toru Ishikawa.

Writing – review & editing: Toru Ishikawa.

## References

[1] Tsochatzis EA Bosch J Burroughs AK. Liver cirrhosis. Lancet. 2014;383:1749–61. 24480518 10.1016/S0140-6736(14)60121-5

[2] Kimer N Wiese S Mo S. Advances in the treatment of portal hypertension in cirrhosis. Expert Rev Gastroenterol Hepatol. 2016;10:961–9. 26982499 10.1586/17474124.2016.1166952

[3] Kockerling D Nathwani R Forlano R. Current and future pharmacological therapies for managing cirrhosis and its complications. World J Gastroenterol. 2019;25:888–908. 30833797 10.3748/wjg.v25.i8.888 PMC6397723

[4] Ge PS Runyon BA. Treatment of patients with cirrhosis. N Engl J Med. 2016;375:767–77. 27557303 10.1056/NEJMra1504367

[5] Garcia-Tsao G Abraldes JG Berzigotti A. Portal hypertensive bleeding in cirrhosis: risk stratification, diagnosis, and management: 2016 practice guidance by the American Association for the Study of Liver Diseases. Hepatology. 2017;65:310–35. 27786365 10.1002/hep.28906

[6] Bosch J. Portal hypertension and cirrhosis: from evolving concepts to better therapies. Clin Liver Dis (Hoboken). 2020;15:S8–12. 32140209 10.1002/cld.844 PMC7050946

[7] Turon F Casu S Hernandez-Gea V. Variceal and other portal hypertension related bleeding. Best Pract Res Clin Gastroenterol. 2013;27:649–64. 24160925 10.1016/j.bpg.2013.08.004

[8] Rockey DC. Management of gastric varices. Gastroenterology. 2001;120:1875–6. 11375970 10.1053/s0016-5085(01)70197-7

[9] Cheng LF Wang ZQ Li CZ. Low incidence of complications from endoscopic gastric variceal obturation with butyl cyanoacrylate. Clin Gastroenterol Hepatol. 2010;8:760–6. 20621678 10.1016/j.cgh.2010.05.019

[10] Kanagawa H Mima S Kouyama H. Treatment of gastric fundal varices by balloon-occluded retrograde transvenous obliteration. J Gastroenterol Hepatol. 1996;11:51–8. 8672742 10.1111/j.1440-1746.1996.tb00010.x

[11] Hirota S Matsumoto S Tomita M. Retrograde transvenous obliteration of gastric varices. Radiology. 1999;211:349–56. 10228513 10.1148/radiology.211.2.r99ma25349

[12] Ryan BM Stockbrugger RW Ryan JM. A pathologic, gastroenterologic, and radiologic approach to the management of gastric varices. Gastroenterology. 2004;126:1175–89. 15057756 10.1053/j.gastro.2004.01.058

[13] Akahoshi T Hashizume M Tomikawa M. Long-term results of balloon-occluded retrograde transvenous obliteration for gastric variceal bleeding and risky gastric varices: a 10-year experience. J Gastroenterol Hepatol. 2008;23:1702–9. 18713295 10.1111/j.1440-1746.2008.05549.x

[14] Ishikawa T Ushiki T Mizuno K. CT-maximum intensity projection is a clinically useful modality for the treatment of gastric varices. World J Gastroenterol. 2005;11:7515–9. 16437726 10.3748/wjg.v11.i47.7515 PMC4725171

[15] Chikamori F Shibuya S Takase Y. Transjugular retrograde obliteration for gastric varices. Abdom Imaging. 1996;21:299–303. 8661570 10.1007/s002619900068

[16] Saad WE Sabri SS. Balloon-occluded retrograde transvenous obliteration (BRTO): technical results and outcomes. Semin Intervent Radiol. 2011;28:333–8. 22942551 10.1055/s-0031-1284460 PMC3312162

[17] Philips CA Ahamed R Rajesh S. Beyond the scope and the glue: update on evaluation and management of gastric varices. BMC Gastroenterol. 2020;20:361. 33126847 10.1186/s12876-020-01513-7 PMC7602314

[18] Nardelli S Riggio O Gioia S. Spontaneous porto-systemic shunts in liver cirrhosis: clinical and therapeutical aspects. World J Gastroenterol. 2020;26:1726–32. 32351289 10.3748/wjg.v26.i15.1726 PMC7183860

[19] Ohnishi K Sato S Saito M. Clinical and portal hemodynamic features in cirrhotic patients having a large spontaneous splenorenal and/or gastrorenal shunt. Am J Gastroenterol. 1986;81:450–5. 3518409

[20] Park JG Tak WY Park SY. Effects of branched-chain amino acid (BCAA) supplementation on the progression of advanced liver disease: a Korean nationwide, multicenter, prospective, observational, cohort study. Nutrients. 2020;12:1429. 10.3390/nu12051429 PMC7284598 32429077

[21] Wang JY Bajaj JS Wang JB. Lactulose improves cognition, quality of life, and gut microbiota in minimal hepatic encephalopathy: a multicenter, randomized controlled trial. J Dig Dis. 2019;20:547–56. 31448533 10.1111/1751-2980.12816

[22] Riggio O Efrati C Catalano C. High prevalence of spontaneous portal-systemic shunts in persistent hepatic encephalopathy: a case-control study. Hepatology. 2005;42:1158–65. 16250033 10.1002/hep.20905

[23] Saad WE. Portosystemic shunt syndrome and endovascular management of hepatic encephalopathy. Semin Intervent Radiol. 2014;31:262–5. 25177088 10.1055/s-0034-1382795 PMC4139430

[24] Lunderquist A Vang J. Transhepatic catheterization and obliteration of the coronary vein in patients with portal hypertension and esophageal varices. N Engl J Med. 1974;291:646–9. 4546968 10.1056/NEJM197409262911303

[25] Ishikawa T Imai M Ko M. Percutaneous transhepatic obliteration and percutaneous transhepatic sclerotherapy for intractable hepatic encephalopathy and gastric varices improve the hepatic function reserve. Biomed Rep. 2017;6:99–102. 28123716 10.3892/br.2016.811 PMC5244787

[26] Maddison FE. Embolic therapy of hypersplenism. Invest Radiol. 1973;8:280–1.

[27] Spigos DG Jonasson O Mozes M. Partial splenic embolization in the treatment of hypersplenism. AJR Am J Roentgenol. 1979;132:777–82. 107745 10.2214/ajr.132.5.777

[28] Shimizu H Takatsuka K Yoshida A. Partial splenic embolization reverses insulin resistance in patients with liver cirrhosis. Intern Med. 2009;48:747–51. 19443968 10.2169/internalmedicine.48.1649

[29] Yoshida H Mamada Y Taniai N. Partial splenic embolization. Hepatol Res. 2008;38:225–33. 18034810 10.1111/j.1872-034X.2007.00302.x

[30] Bárcena R Moreno A Foruny JR. Improved graft function in liver-transplanted patients after partial splenic embolization: reversal of splenic artery steal syndrome? Clin Transplant. 2006;20:517–23. 16842531 10.1111/j.1399-0012.2006.00516.x

[31] Ishikawa T Sasaki R Nishimura T. Short-term effects of hepatic arterial buffer responses induced by partial splenic embolization on the hepatic function of patients with cirrhosis according to the Child-Pugh classification. Intern Med. 2021;60:1331–42. 33281164 10.2169/internalmedicine.6267-20 PMC8170249

[32] N’Kontchou G Seror O Bourcier V. Partial splenic embolization in patients with cirrhosis: efficacy, tolerance and long-term outcome in 32 patients. Eur J Gastroenterol Hepatol. 2005;17:179–84. 15674095 10.1097/00042737-200502000-00008

[33] Talwar A Gabr A Riaz A. Adverse events related to partial splenic embolization for the treatment of hypersplenism: a systematic review. J Vasc Interv Radiol. 2020;31:1118–31.e6. 32014400 10.1016/j.jvir.2019.08.015

[34] Hayashi H Beppu T Okabe K. Risk factors for complications after partial splenic embolization for liver cirrhosis. Br J Surg. 2008;95:744–50. 18412294 10.1002/bjs.6081

[35] Ishikawa T Imai M Okoshi M. Cone beam versus conventional computed tomographic angiography volume measurement in partial splenic embolization. Medicine (Baltim). 2019;98:e14312. 10.1097/MD.0000000000014312 PMC6380856 30702608

[36] Peck-Radosavljevic M. Thrombocytopenia in chronic liver disease. Liver Int. 2017;37:778–93. 27860293 10.1111/liv.13317

[37] Clavien PA Camargo CA Jr Croxford R. Definition and classification of negative outcomes in solid organ transplantation. Application to liver transplantation. Ann Surg. 1994;220:109–20. 8053733 10.1097/00000658-199408000-00002 PMC1234350

[38] Ishikawa T Kubota T Horigome R. Concurrent partial splenic embolization with transcatheter arterial chemoembolization for hepatocellular carcinoma can maintain the hepatic functional reserve. Hepatol Res. 2014;44:1056–61. 23941627 10.1111/hepr.12222

[39] Hadduck TA McWilliams JP. Partial splenic artery embolization in cirrhotic patients. World J Radiol. 2014;6:160–8. 24876920 10.4329/wjr.v6.i5.160 PMC4037542

[40] Koconis KG Singh H Soares G. Partial splenic embolization in the treatment of patients with portal hypertension: a review of the English language literature. J Vasc Interv Radiol. 2007;18:463–81. 17446537 10.1016/j.jvir.2006.12.734

[41] Corazza GR Ginaldi L Zoli G. Howell-Jolly body counting as a measure of splenic function. A reassessment. Clin Lab Hematol. 1990;12:269–75. 10.1111/j.1365-2257.1990.tb00037.x 2125541

[42] Ishikawa T Kubota T Horigome R. Prevalence of Howell-Jolly bodies caused by partial splenic embolization for portal hypertension. Intern Med. 2013;52:1765–8. 23955609 10.2169/internalmedicine.52.0407

[43] Rösch J Hanafee WN Snow H. Transjugular portal venography and radiologic portacaval shunt: an experimental study. Radiology. 1969;92:1112112–1114. 10.1148/92.5.1112 5771827

[44] Colapinto RF Stronell RD Gildiner M. Formation of intrahepatic portosystemic shunts using a balloon dilatation catheter: preliminary clinical perience. AJR Am J Roentgenol. 1983;140:709–14. 6601376 10.2214/ajr.140.4.709

[45] Richter GM Noeldge G Palmaz JC. Transjugular bypass of intrahepatic portacaval stent: preliminary clinical results. Radiology. 1990;174:1027–30. 2305084 10.1148/radiology.174.3.174-3-1027

[46] Yamada R. Experience of transjugular intrahepatic portosystemic shunt (TIPS). J Jpn Radiol Soc. 1992;52:1328–30. 1437540

[47] Zhang L Li Q Makamure J. Transjugular intrahepatic portosystemic shunt for hepatic sinusoidal obstruction syndrome associated with consumption of Gynura segetum. BMC Gastroenterol. 2021;21:26. 33423668 10.1186/s12876-021-01599-7 PMC7798314

[48] Liu J Wehrenberg-Klee EP Bethea ED. Transjugular intrahepatic portosystemic shunt placement for portal hypertension: meta-analysis of safety and efficacy of 8 mm vs. 10 mm stents. Gastroenterol Res Pract. 2020;2020:9149065. 33123192 10.1155/2020/9149065 PMC7586157

[49] Rajesh S George T Philips CA. Transjugular intrahepatic portosystemic shunt in cirrhosis: an exhaustive critical update. World J Gastroenterol. 2020;26:5561–96. 33088154 10.3748/wjg.v26.i37.5561 PMC7545393

[50] Barbier CB Rorsman F Eriksson LG. Placement of a transjugular intrahepatic portosystemic shunt in addition to recanalization of acute and chronic portomesenteric vein occlusions - a retrospective evaluation. Acta Radiol Open. 2020;9:1–7. 10.1177/2058460120964074 PMC7557710 33110628

[51] Qi X Han G. Transjugular intrahepatic portosystemic shunt in the treatment of portal vein thrombosis: a critical review of literature. Hepatol Int. 2012;6:576–90. 26201472 10.1007/s12072-011-9324-5 PMC7101972

[52] Sommer CM Gockner TL Stampfl U. Technical and clinical outcome of transjugular intrahepatic portosystemic stent shunt: bare metal stents (BMS) versus Viatorr stent-grafts (VSG). Eur J Radiol. 2012;81:2273–80. 21784593 10.1016/j.ejrad.2011.06.037

[53] Casado M Bosch J García-Pagán JC. Clinical events after transjugular intrahepatic portosystemic shunt: correlation with hemodynamic findings. Gastroenterology. 1998;114:1296–303. 9609767 10.1016/s0016-5085(98)70436-6

[54] Weber CN Nadolski GJ White SB. Long-term patency and clinical analysis of expanded polytetrafluoroethylene-covered transjugular intrahepatic portosystemic shunt stent grafts. J Vasc Interv Radiol. 2015;26:1257–65. 25990133 10.1016/j.jvir.2015.04.005

[55] Jung HS Kalva SP Greenfield AJ. TIPS: comparison of shunt patency and clinical outcomes between bare stents and expanded polytetrafluoroethylene Stent-Grafts. J Vasc Intervent Radiol. 2009;20:180–5. 10.1016/j.jvir.2008.11.005 19097918

[56] Fidelman N Kwan SW LaBerge JM. The transjugular intrahepatic portosystemic shunt: an update. Am J Roentgenol. 2012;199:746–55. 22997364 10.2214/AJR.12.9101

[57] García-Pagán JC Caca K Bureau C. Early TIPS (transjugular intrahepatic portosystemic shunt) Cooperative Study Group. Early use of TIPS in patients with cirrhosis and variceal bleeding. N Engl J Med. 2010;362:2370–9. 20573925 10.1056/NEJMoa0910102

[58] Lim J Kim HI Kim E. Variceal bleeding is aggravated by portal venous invasion in hepatocellular carcinoma: a matched nested case-control study. BMC Cancer. 2021;21:11. 33402105 10.1186/s12885-020-07708-1 PMC7786454

[59] Yamakado K Tanaka N Nakatsuka A. Clinical efficacy of portal vein stent placement in patients with hepatocellular carcinoma invading the main portal vein. J Hepatol. 1999;30:660–8. 10207808 10.1016/s0168-8278(99)80197-4

[60] Haddad MM Fleming CJ Thompson SM. Comparison of bleeding complications between transsplenic versus transhepatic access of the portal venous system. J Vasc Interv Radiol. 2018;29:1383–91. 30174158 10.1016/j.jvir.2018.04.033

[61] Yamakado K Nakatsuka A Tanaka N. Malignant portal venous obstructions treated by stent placement: significant factors affecting patency. J Vasc Interv Radiol. 2001;12:1407–15. 11742015 10.1016/s1051-0443(07)61699-6

[62] Ishikawa T Kubota T Abe H. Percutaneous transhepatic portal vein stent placement can improve the prognosis of patients with hepatocellular carcinoma with portal vein tumor thrombosis. Hepatogastroenterology. 2014;61:413–6. 24901152

[63] Pillai AK Andring B Patel A. Portal hypertension: a review of portosystemic collateral pathways and endovascular interventions. Clin Radiol. 2015;70:1047–59. 26188844 10.1016/j.crad.2015.06.077

[64] Hashizume M Tanoue K Akahoshi T. Laparoscopic splenectomy: the latest modern technique. J Gastroenterol Hepatol. 2002;17:77–80. 10370620

